# Prediction of suboptimal cytoreductive surgery in patients with advanced ovarian cancer based on preoperative and intraoperative determination of the peritoneal carcinomatosis index

**DOI:** 10.1186/s12957-018-1339-0

**Published:** 2018-02-23

**Authors:** Antoni Llueca, Anna Serra, Isabel Rivadulla, Luis Gomez, Javier Escrig, MUAPOS working group (Multidisciplinary Unit of Abdominal Pelvic Oncology Surgery)

**Affiliations:** 1 Deparment of Obstetrics and Gynecology, Av Benicasim s/n, 12004 Castellón, Spain; 2 Department of General Surgery, Castellón, Spain; 3 Multidisciplinary Unit of Abdominal Pelvic Oncology Surgery (MUAPOS), University General Hospital of Castellon, Av Benicasim s/n, 12004 Castellón, Spain; 4 Department of Medicine, University Jaume I (UJI), Av Benicasim s/n, 12004 Castellón, Spain

**Keywords:** Advanced ovarian cancer, Cytoreductive surgery, Peritoneal carcinomatosis index

## Abstract

**Background:**

The peritoneal carcinomatosis index (PCI) can be used to quantify the tumor burden in patients with advanced ovarian cancer. The aim of the present study was to establish a predictive model for suboptimal cytoreductive surgery (SCS) (residual tumor of > 1 cm) using preoperative and intraoperative determination of the PCI.

**Methods:**

In total, 110 consecutive patients treated for advanced ovarian cancer during a 4-year period in our institution were assessed. Eighty of these patients were eligible for primary debulking surgery and thus included in the present study. All data were prospectively collected and retrospectively evaluated. We determined the PCI both preoperatively and intraoperatively and assessed postoperative complications.

**Results:**

A PCI of > 20 was the best cut-off with which to predict a risk of SCS among all three diagnostic techniques assessed in this study (computed tomography, laparoscopy, and laparotomy). Intraoperative PCI determination was associated with the lowest risk of false negatives for SCS when detecting a PCI of < 20. The combination of preoperative computed tomography and laparoscopy, when both techniques predicted SCS, was associated with the lowest risk of false positives for SCS when detecting a PCI of > 20.

**Conclusion:**

The combination of computed tomography and laparoscopy to obtain the PCI can help to determine which patients with advanced ovarian cancer are suitable for primary debulking surgery and which should undergo neoadjuvant chemotherapy.

## Background

Ovarian cancer is a major cause of gynecologic cancer-related death in women despite treatment advances during the past few decades. Ovarian cancer accounts for 5% of all cancers among women and causes more deaths than any other female genital tract cancer. Approximately 8 in 100,000 women per year in Spain will develop ovarian cancer [1].

Approximately 70 to 80% of ovarian cancers are diagnosed at an advanced stage (stage III and IV). For this group of patients, the 5-year survival rate is lower than 20 to 30% [1]. The gold standard treatment of ovarian cancer has remained the same over the last few decades, consisting of primary cytoreductive surgery to resect as much diseased tissue as possible followed by platinum-based chemotherapy [2].

Surgery is considered essential for treatment of advanced epithelial ovarian cancer (AOC). In general, cytoreductive surgery is initially performed for AOC, including stage IV, when distant metastatic lesions develop and do not influence survival in the short term. The ultimate goal of cytoreductive surgery is removal of the entire tumor burden to achieve either complete removal of the tumor upon visual inspection (complete cytoreductive surgery, CCS) or a residual tumor of < 1 cm (optimal cytoreductive surgery, OCS). Only these two surgical outcomes will improve survival [3].

Intraperitoneal spread of ovarian cancer is the most typical presentation of stage III and IV ovarian cancer. Various assessment tools in the field of surgical oncology have been described to objectively evaluate and quantify the tumor burden in patients with these AOC stages. One of the most frequently used tools is the peritoneal carcinomatosis index (PCI) for peritoneal carcinomatosis of all types, first described by Sugarbaker and Jablonski [4]. Fagotti et al. [5], Aletti et al. [6], and Zivanovic et al. [7] have also described other assessment tools specifically for ovarian cancer. These assessment tools are based on tumor size and/or location within the peritoneal cavity.

One series regarding debulking or cytoreductive surgery for treatment of AOC demonstrated cytoreduction rates of 15 to 85%. In most such reports, when the OCS is > 50%, upper abdominal surgery (UAS) is employed [8]. However, these extensive surgical procedures to achieve complete cytoreduction are associated with postoperative morbidity rates ranging from 11.0 to 67.0% and postoperative mortality rates ranging from 0.0 to 6.7% [9].

Based on these data, the gynecologic oncologist must decide between efficacy and safety when performing these procedures. Neoadjuvant chemotherapy (NACT) and interval debulking surgery are valid alternatives in patients for whom high complication rates are expected (e.g., severe comorbidities, poor performance status) or when unresectable disease is identified prior to surgery. Therefore, one of the key points of surgical treatment is to predict whether the surgical team will be capable of performing OCS. In this context, some predictive cytoreduction models have been described during the last few years [5].

The aim of this study was to establish a predictive model for suboptimal cytoreductive surgery (SCS) based on our experience with preoperative and intraoperative determination of the PCI by computed tomography (CT) and laparoscopy at the Multidisciplinary Unit for Abdominal Pelvic Oncology Surgery (MUAPOS).

## Methods

In total, 110 consecutive patients with AOC were treated at the MUAPOS of the University General Hospital of Castellon (Spain) from January 2013 to December 2016. The risk of suboptimal cytoreductive surgery was evaluated with the radiologic-laparoscopic criteria for unresectability (RLCU) (Table 1) in the preoperative studies. Age and health status were also taken into account. All procedures were carried out by the same surgical team.

**Table 1:** Radiologic-laparoscopic criteria for unresectability

CT scan	Lung metastasis Hepatic metastasis in three or more hepatic segments Severe hepatic pedicle involvement Progression after NACT
Diagnostic laparoscopy	Diffuse serous small bowel disease

*CT* computed tomography, *NACT* neoadjuvant chemotherapy

The PCI was determined in all patients by preoperative laparoscopy (49 patients) and/or thoracoabdominal CT (80 patients). To quantify the radiological PCI, the largest tumor implant was chosen in the assessed region, and a score was assigned from 0 to 3 points. The sum of the score assigned to each region results in the radiological PCI which varies between 1 and 39 points as the operative index. The PCI was calculated before and during surgery and was categorized into three ordinal levels: 1–10, 11–20, and > 20. The best PCI cut off for SCS was calculated with a ROC curve [10]. All patients’ clinical and pathological characteristics, surgical procedures, and residual disease at surgery were prospectively collected and retrospectively analyzed for the purpose of this study.

With respect to the presence of a residual tumor at the end of surgery, CCS was defined as no residual macroscopic tumor, OCS was defined as a residual tumor of < 1 cm, and SCS was defined as a residual tumor of > 1 cm. Postoperative complications were described according to the Clavien–Dindo classification [11]. Grade ≥ II complications were considered major complications. The relationship between major complications and visceral resections was analyzed with a cumulative sum (CUSUM) graph of cumulative risk proposed by Royston [12]. All specimens were collected and labeled relating the PCI areas. The study was approved by the ethics committee of our institution.

The sensitivity (SEN), specificity (SPC), positive predictive value (PPV), and negative predictive value (NPV) were calculated for each investigated parameter. SEN was defined as the number of patients with a residual tumor of > 1 cm at surgery (SCS) who were correctly identified (true positives) divided by the total number of patients who underwent SCS (true positives + false negatives). SPC was defined as the number of patients with a residual tumor of < 1 cm at surgery (CCS + OCS) who were properly identified (true negatives) divided by the total number of patients who underwent CCS + OCS (true negatives + false positives). The PPV was calculated as the number of true positives divided by the number of total positives (true positives + false positives). The NPV was defined as the number of true negatives divided by the number of total negatives (false negatives + true negatives).

Spearman’s rho correlation coefficient was calculated to analyze the association between quantitative and ordinal variables. STATA v12 software was used for statistical analysis. A *p* value of < 0.05 was considered statistically significant.

## Results

From January 2013 to December 2016, 110 patients suspected to have AOC were treated at the MUAPOS at the University General Hospital of Castellon (Spain). Among them, 80 patients where eligible for primary debulking surgery (PDS) and were included in this study. None of these patients met our radiologic-laparoscopic criteria for unresectability (RLCU) (Table 1). Thirty patients (27.2%) who underwent neoadjuvant chemotherapy followed by interval debulking surgery were excluded from the study.

The clinicopathologic characteristics of all patients are summarized in Table 2. Most patients presented with serous (55%), FIGO stage IIIC (69%) epithelial ovarian cancer. At laparotomy, CCS and OCS were achieved in 64 (80.0%) and 5 (6.2%) patients, respectively, while SCS was achieved in the remaining 11 (13.8%) patients.

**Table 2:** Clinicopathologic characteristics of all patients

Characteristics	Nr.	(%)
All cases	80	
Age, median (range), years	59 (30–84)	
FIGO stage		
III c	55	69
IV	25	31
Ascites		
Yes	22	27.5
No	58	72.5
Histology		
Serous	44	55
Endometrioid/clear cells	12	15
Mucinous	8	10
Other adenocarcinoma	16	20
PCI, median (range)	12 (2–35)	
PCI 1–10	34	42.5
PCI 11–20	25	31.2
PCI > 20	21	26.2
Duration of surgery, median (range)	360 (60–638)	
Residual tumor		
CCS	64	80
OCS	5	6.2
SCS	11	13.8

*PCI* peritoneal carcinomatosis index, *CCS* complete cytoreductive surgery, *OCS* optimal cytoreductive surgery, *SCS* suboptimal cytoreductive surgery

The area under the receiver operating characteristic curve showed that the best cut-off for predicting a risk of SCS was a PCI of > 20 for the three diagnostic techniques (CT, laparoscopy, and laparotomy). Preoperative CT predicted possible SCS (PCI > 20) in nine (11%) patients. In eight (16%) patients, preoperative laparoscopy predicted possible SCS (PCI > 20). In 21 (26%) patients, PCI determination at the start of surgery predicted possible SCS (PCI > 20). Table 3 shows the diagnostic parameters of the three techniques in the prediction of SCS risk.

**Table 3:** Diagnostic parameters for SCS by technique

	SEN (95% CI)	SPC (95% CI)	PPV^a^ (95% CI)	NPV^a^ (95% CI)
CT scan	27% (6–61)	91% (82–97)	33% (8–70)	89 (79–95)
Laparoscopy	38% (9–76)	88% (74–96)	33% (6–73)	90 (76–97)
Laparotomy	73% (39–94)	81% (70–90)	38% (18–62)	95% (86–99)
CT plus laparoscopy^b^	38% (14–69)	98% (87–99)	75% (27–98)	91% (82–96)

*SCS* suboptimal cytoreductive surgery, *95%CI* 95% confidence interval, *SEN* sensitivity, *SPC* specificity, *PPV* positive predictive value, *NPV* negative predictive value, *CT* computed tomography

^a^For an SCS prevalence of 13.8%

^b^Criteria for SCS: CT plus laparoscopy positive for SCS if both show a PCI of > 20

Thus, the diagnostic technique with the lowest risk of false negatives for SCS (27%) when detecting a PCI of < 20 was intraoperative PCI determination. The diagnostic technique with the lowest risk of false positives for SCS (2%) when detecting a PCI of > 20 was the combination of preoperative CT and laparoscopy when both techniques predicted SCS.

**Fig. 1:**
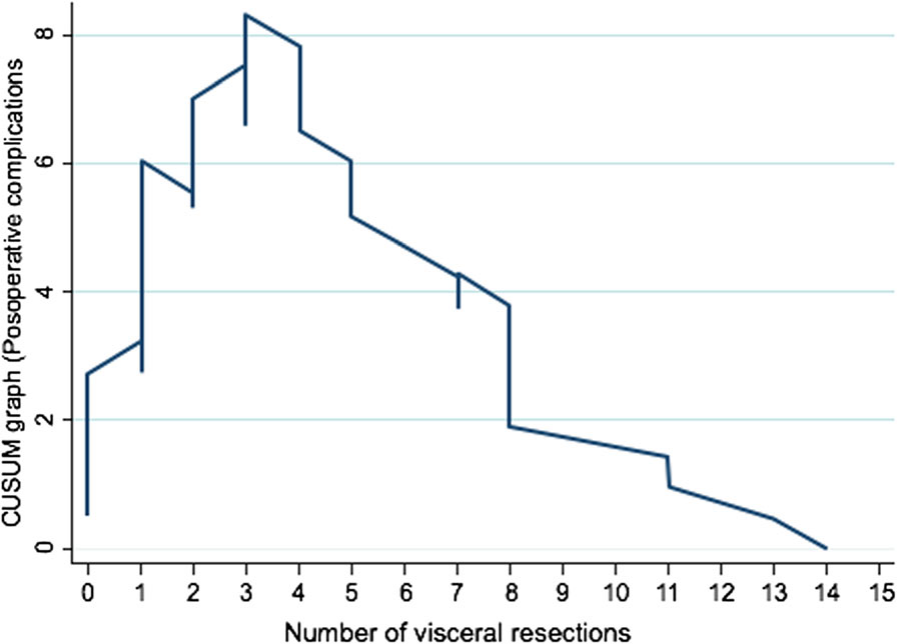
CUSUM graph relationship between visceral resections and postoperative complications

The surgical procedures performed in all series included abdominal and pelvic peritonectomy in 54 (67%) patients, rectosigmoidectomy in 35 (43%), and large bowel resection in 40 (50%). Among them, UAS was required in 57 (71%) patients, including diaphragmatic peritonectomy in 40 (50%), distal pancreatectomy in 8 (10%), splenectomy in 23 (28.7%), and liver resection in 9 (11%). Major postoperative complications (Clavien–Dindo grade II–IV) were found in 45 (56%) patients. Grade III to IV complications were found in 33 (37%) patients, with a higher incidence at a PCI of > 10 (*p* < 0.001). The highest rate of postoperative complications were found when PCI > 20 (*p* < 0.01). The number of visceral resections was correlated with the intraoperative PCI (*p* < 0.001). The CUSUM graph [11] of the cumulative risk of major postoperative complications by the number of visceral resections showed that the risk of postoperative complications progressively increased from three visceral resections (*p* < 0.001) until a maximum level was maintained from eight visceral resections (Fig. 1). The 90-day postoperative mortality rate was 3.7% (3 patients) and was mainly related to the number of visceral resections (*p* = 0.009). The number of visceral resections was correlated with the intraoperative PCI (*p* < 0.001).

## Discussion

Prediction of surgical outcomes in patients with AOC is a current surgical dilemma. Surgery remains the cornerstone of treatment for advanced disease, but it is not applicable to all patients. NACT remains the best option for patients who are not surgical candidates [13]. Vergote et al. [14] reported similar survival with PDS and with NACT in stages III and IV ovarian cancer but these results received multiple criticisms because of the design of the study with an overall rate of complete cytoreduction of 19.4% at PDS; actually, these results are not in the general philosophy of treatment of this disease.

Intraperitoneal spread is the most typical presentation of stage III and IV ovarian cancer. Unfortunately, the FIGO system fails in characterization of tumor burden and in describing anatomical regions affected. The PCI describes precisely the anatomic regions affected and objectively quantifies the tumor volume. Tumor load is an important prognostic factor and should guide the treatment strategy, patients with stage IIIc and less extensive metastatic tumors had higher survival with PDS while patients with stage IV disease and large metastatic tumors had higher survival with NACT and IDS [15]. In our data, 11 patients had SCS, 7 (64%) cases were FIGO stage IV, and more than 50% of them have a PCI > 20. With the present model, we should send them to NACT.

The best method for choosing between PDS and NACT is still not universally defined. In this paper, we describe a combination of methods that can be useful to predict the outcome of AOC or SCS surgery based on preoperative quantification of the PCI.

The accuracies of CT and diagnostic laparoscopy in preoperative quantification of the PCI were similar in the present study. CT might be better able to quantify the tumor burden affecting strategic anatomic landmarks, which is a contraindication for initial surgery. At the same time, laparoscopy allows for visualization of diffuse serous small bowel disease, which is another contraindication for PDS (Table 1).

Prediction of OCS or SCS remains a key factor in AOC treatment. Some authors have described CT as the best method with which to predict the surgical outcome. In fact, the accuracy of CT in detecting tumor implants has ranged from 60 to 90% in some series (Table 4). Carcinomatosis affecting the pelvic sidewall and extensive upper abdominal disease with tumor involvement of the diaphragm, liver, porta hepatis, and lesser omentum have traditionally been considered reasons to abandon PDS and instead perform NACT [16–20].

**Table 4:** Studies using CT to predict surgical outcome

Study	*n*	OCS (%)	SEN (%)	SPC (%)	PPV (%)	NPV (%)
Nelson	42	69	92	71	67	94
Meyer	28	57	58	100	100	55
Bristow	41	49	100	85	87	100
Dowdy	87	71	64	81	57	85
Llueca	80	86	91	27	89	33

*CT* computed tomography, *OCS* optimal cytoreductive surgery, *SEN* sensitivity, *SPC* specificity, *PPV* positive predictive value, *NPV* negative predictive value

In the present study, UAS was required in 57 (71%) patients; this is not currently considered a contraindication for PDS in centers with an aggressive surgical protocol and higher rates of OCS [21, 22]. The results shown in Table 5 reflect the difficulty of reproducing different predictive models in different patient populations with different surgeons. With our 86% rate of OCS, it is very difficult to obtain a high PPV for SCS by radiology or another technique (Table 5). OCS varies among institutions, and surgeons’ efforts are influenced by their surgical skills, training, and policy toward this type of surgery in their institution. This demonstrates the surgeon-dependent nature of the cytoreductive surgery outcome. The results of the preoperative CT evaluation depend on the results of OCS; higher OCS rates are associated with lower PPVs on the CT evaluation for SCS.

**Table 5:** Predictive values depending on prevalence of SCS (CT + laparoscopy)

SCS: prevalence	SCS: PPV	SCS: NPV
10%	68% (17–98)	93% (85–98)
20%	83% (40–99)	86% (76–93)
30%	89% (55–99)	79% (67–88)
40%	93% (65–99)	70% (58–81)
50%	95% (72–99)	61% (48–73)

For sensitivity of 38% and specificity of 98%

*SCS* suboptimal cytoreductive surgery, *CT* computed tomography, *PPV* positive predictive value, *NPV* negative predictive value

Because of difficulties in predicting SCS using only imaging techniques, some investigators have explored laparoscopy for the prediction of resectability in patients with AOC. Fagotti et al. [5] described a laparoscopic model based on a scoring system from 0 to 12 for progressive disease. The authors reported an overall OCS rate of 67%. This model has a good PPV for OCS and an acceptable NPV for scores of < 2 and > 8. For scores of 2 to 8, however, the surgeon encountered a variable rate of unnecessary exploration. External validation of this score was performed by Brun et al., who reported that an OCS rate of 69% had an SEN, SPC, PPV, and NPV of 46, 89, 89, and 44%, respectively, with a decreased accuracy of 60% [23]. Petrillo et al. [24] recently increased their OCS rate to 80% by introducing UAS into their procedures. Once again, the key point is the relationship between surgeon-related factors and the surgical outcome; a better-trained surgical team will produce a lower failure rate in terms of the PPV for OCS, and this may vary with time. Based on our results, the best individual method with which to predict the outcome of AOC surgery is the first inspection during the initial laparotomy. However, we obtained better results when combining preoperative CT and laparoscopy for OCS when they both revealed a PCI of < 20 (SEN, 98%; SPC, 38%; PPV, 91%; and NPV, 75%). This means that only 9% (1 − PPV) of patients are at risk for unnecessary exploration.

Moreover, according to our 13.8% prevalence of SCS and with a PCI cut-off of 20 (Table 3), the best rate of a correct diagnosis with SCS (PPV) was obtained by the combination of preoperative CT and laparoscopy (75%). However, the PPV and NPV depend on the SCS rate achievable by the surgical team, assuming the same SEN and SPC as those obtained in the present study. Table 5 shows the possible variations in PPV and NPV depending on the SCS rate.

From Table 5, we can deduce that the final reliability (PPV) in the prediction of SCS according to our criteria and PCI cut-off may be acceptable for SCS rates of ≥ 20%. This is extendable to any predictive diagnostic model for SCS regardless of the criteria used to predict SCS because predictive values depend on the prevalence of SCS for a specific SEN and SPC (those shown in the present study). Thus, only one model that offers a very high SEN and SPC will derive acceptable predictive values even with a low prevalence of SCS.

Visceral resections were performed in 85% of patients. A CUSUM graph of the cumulative risk of major complications by the number of visceral resections was created to evaluate the relationship between visceral resections and complications. This graph shows the cumulative sum of observed minus expected postoperative complications (expected = 56%) by the number of visceral resections, which proves a clear relationship between the two factors (Fig. 1). Some authors have reported that an increased number of visceral resections increases the morbidity [25]. In the present study, the highest rate of complications occurred in association with eight or more visceral resections, and three or more visceral resections were performed in all patients with a PCI of > 20. In our results, 21 patients had PCI > 20, 11 with SCS, and 10 with OCS but the last ones suffered a high rate of complications. Well-trained surgeons can perform incredible surgeries even in high PCI, but based on our results, we demonstrate that PCI is correlated with the number of complications. We believe that higher PCI (> 20) should go to NACT in order to assure the security of patients.

**Fig. 2:**
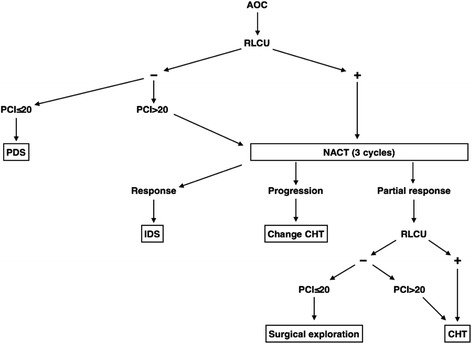
MUAPOS guide to manage AOC. *MUAPOS* Multidisciplinary Unit for Abdominal Pelvic Oncology Surgery, *AOC* advanced epithelial ovarian cancer, *PDS* primary debulking surgery, *IDS* interval debulking surgery, *CHT* chemotherapy, *NACT* neoadjuvant chemotherapy, *RLCU* radiologic-laparoscopic criteria for unresectability

It is possible that a multimodal model may overcome the individual limitations of the herein discussed methods to predict OCS or SCS. Our management and treatment protocol for AOC balances both effectiveness and patient safety (Fig. 2).

This multimodal method of managing the surgical approach to AOC treatment is based on the results of the present study, and we combine CT and laparoscopy to determine which patients are suitable for PDS and which should undergo NACT with an acceptable OCS rate and minimal postoperative complications. Other individual characteristics such as the performance status, age, and nutritional status are also taken into account before making the final decision [26].

## Conclusion

In conclusion, every individual method employed to predict the surgical outcome in patients with AOC has its own limitations; perhaps a multimodal model may overcome these limitations. Nevertheless, due to the surgeon-dependent nature of this disease, any model employed should be individualized for the surgical team and should be evolutional over time as the surgeons’ effectiveness improves.

## Abbreviations

AOC: Advanced epithelial ovarian cancer
CCS: Complete cytoreductive surgery
CT: Computed tomography
CUSUM: Cumulative sum
MUAPOS: Multidisciplinary Unit of Abdominal Pelvic Oncology Surgery
NACT: Neoadjuvant chemotherapy
NPV: Negative predictive value
OCS: Optimal cytoreductive surgery
PCI: Peritoneal carcinomatosis index
PDS: Primary debulking surgery
PPV: Positive predictive value
RLCU: Radiologic-laparoscopic criteria for unresectability
SCS: Suboptimal cytoreductive surgery
SEN: Sensitivity
SPC: Specificity
UAS: Upper abdominal surgery
UJI: University Jaume I
